# BSCN: bidirectional symmetric cascade network for retinal vessel segmentation

**DOI:** 10.1186/s12880-020-0412-7

**Published:** 2020-02-18

**Authors:** Yanfei Guo, Yanjun Peng

**Affiliations:** 1 College of Information Science and Engineering,Shandong University of Science and Technology, Shandong, Qingdao 266590 China; 2 Shandong Province Key Laboratory of Wisdom Mining Information Technology, Shandong University of Science and Technology, Shandong, Qingdao 266590 China

**Keywords:** Retinal vessel segmentation, Bidirectional symmetric cascade network, Specific diameter scale, Dense dilated convolution, Scale detection

## Abstract

**Background:**

Retinal blood vessel segmentation has an important guiding significance for the analysis and diagnosis of cardiovascular diseases such as hypertension and diabetes. But the traditional manual method of retinal blood vessel segmentation is not only time-consuming and laborious but also cannot guarantee the accuracy and efficiency of diagnosis. Therefore, it is especially significant to create a computer-aided method of automatic and accurate retinal vessel segmentation.

**Methods:**

In order to extract the blood vessels’ contours of different diameters to realize fine segmentation of retinal vessels, we propose a Bidirectional Symmetric Cascade Network (BSCN) where each layer is supervised by vessel contour labels of specific diameter scale instead of using one general ground truth to train different network layers. In addition, to increase the multi-scale feature representation of retinal blood vessels, we propose the Dense Dilated Convolution Module (DDCM), which extracts retinal vessel features of different diameters by adjusting the dilation rate in the dilated convolution branches and generates two blood vessel contour prediction results by two directions respectively. All dense dilated convolution module outputs are fused to obtain the final vessel segmentation results.

**Results:**

We experimented the three datasets of DRIVE, STARE, HRF and CHASE_DB1, and the proposed method reaches accuracy of 0.9846/0.9872/0.9856/0.9889 and AUC of 0.9874/0.9941/0.9882/0.9874 on DRIVE, STARE, HRF and CHASE_DB1.

**Conclusions:**

The experimental results show that compared with the state-of-art methods, the proposed method has strong robustness, it not only avoids the adverse interference of the lesion background but also detects the tiny blood vessels at the intersection accurately.

## Background

A new study published in the British "Lancet Global Health" predicts that if the treatment of eye diseases is not improved by better funding, the number of blind people worldwide will increase to 115 million by 2050, 2.2 times more than the current 36 million [1]. In fact, Retinal vessel disease in the fundus is one of the vital causes of blindness and many can be prevented in advance by fundus retinal examination among a large number of irreversible blinding diseases.

The retinal vessel is the only clear blood vessel that can be observed by non-invasive means. Current medical research shows that the abnormality of retinal vascular is not only manifested in ophthalmic diseases such as glaucoma and cataract but also directly related to the severity of cardiovascular diseases such as hypertension, coronary heart disease, diabetes, atherosclerosis [2]. The morphological structure of retinal blood vessels in fundus can reflect the condition of the blood vessels in the eyes and around the body. It can predict, diagnose and prevent cardiovascular diseases effectively by analyzing the retinal images [3]. Therefore, the research of retinal vessel segmentation technology is helpful to automatically and quickly obtain the morphological structure of blood vessels in retinal images and has extremely crucial clinical significance and practical value for assistant diagnosis and treatment of various related diseases [4].

**Fig. 1:**
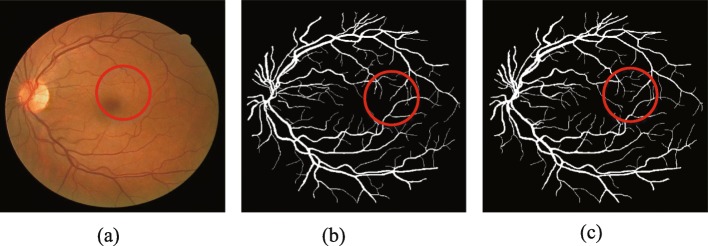
Comparison of two manual segmentation results of the retinal. **a** original image **b** 1st manual label **c** 2nd manual label

Retinal blood vessels are usually segmented manually by ophthalmologists relying on experience in the past. But it is difficult to completely segment retinal blood vessels due to the intricate distribution of vessels [5], the low contrast between vessel and background, and lesion interference and uneven illumination in fundus images. In addition, manual labeling has greater subjectivity. As shown in Fig. 1, the manual segmentation results of the same retinal image by the two experts are not the same. Generally speaking, the traditional manual method of retinal blood vessel segmentation is not only time-consuming and laborious but also cannot guarantee the accuracy and efficiency of diagnosis [6]. Therefore, it is particularly important to create a computer-aided method of automatic and accurate retinal vascular segmentation.

Aiming at the above problems, many researchers proposed to adopt deep learning methods for retinal blood vessel segmentation. However, due to the difference in morphological scale in diameter, tortuosity, branching pattern or angles of the blood vessels, most methods can segment the thick and obvious blood vessels, but cannot segment the tiny blood vessels accurately. In addition, the predicted segmentation results are unsatisfactory when training the different network layers by a general retinal vessel’s ground truth. Because of the difference in receptive fields, the lower layers of the network can obtain more local image information, while the higher layers capture object-level information by a larger receptive field. Different network layers can extract feature information of different scales respectively, so it is not wise to use the same supervision to train different network layers.

In this paper, we innovatively introduce cascaded networks into the vessel segmentation task and propose a Bidirectional Symmetric Cascade Network (BSCN) to segment the retinal vessels more effectively. First, the original fundus image is input into the scale detection module to extract vascular features of different diameter scales, and each scale detection module generates two blood vessel contour prediction maps from the low layer to the high layer and from the high layer to the low layer. The final blood vessel segmentation result is calculated by convolution fusion of the blood vessel contour prediction maps generated by all the intermediate layers at last. To the best of our knowledge, this is the first time to adopt the bidirectional symmetric cascade network for retinal vessel segmentation.

The three creative points of this paper are summarized as follows:

1) This paper creatively transforms retinal vessel segmentation into a multi-scale contour detection problem, improves the VGG16 network, and designs a lightweight network structure of Scale Detection Block (SDB) for retinal vessel segmentation.

2) In order to better capture the richer details of retinal vessels and make full use of the multi-scale features of blood vessels, we propose a Dense Dilated Convolution Module (DDCM). The tiny and blurred blood vessel information is captured by using multiple dilated convolutions of different dilation rates without significantly increasing the network parameters.

3) So as to allow each layer in the CNN to perform specific supervised training and adaptively learn the scale information from each layer, this paper proposes the Bidirectional symmetric cascade network (BSCN) architecture. To achieve multi-scale retinal vessel segmentation, it consists of several DDCM inserted into the SDB constructed by the VGG block. The bidirectional cascading structure allows each layer of the network to focus on learning vessel feature at a specific scale, better optimizing the training process and avoiding computational redundancy.

The organization of the paper is as follows. “Related work” section introduces the related work of retinal blood vessel segmentation. “Methods” section describes the proposed method in detail, including the overall architecture of the bidirectional symmetric cascade network, the scale detection module and the dense dilated convolution module. “Results” section introduces the experimental dataset, environment setup, evaluation metrics and analysis of experimental results. “Discussion” section discusses the advantages of our method over other methods. “Conclusions” section provides a conclusion with the future work plan.

## Related work

### Unsupervised method

The unsupervised segmentation method does not need prior labeling information and extracts the color, texture and other feature representation of the blood vessel. According to different image processing methods, it can be subdivided into the model-based method [7], vascular tracking [8], matched filtering [9] and mathematical morphology [10]. Zhao et al. [11] applied level set and region growth to segment retinal blood vessel. Nayebifar et al. [12] used particle filtering to track the retinal vessel paths for automatic blood vessel segmentation. The method can describe the structure of the vascular network comprehensively, and the adaptability is good, but the computation amount is large and depends on the selection of the initial seed point and direction. Moreover, the branch points of the blood vessel and the low-contrast blood vessels cannot be segmented effectively. Azzopardi et al. [13] introduced B-COSFIRE filters to automatic vessel trees segmentation. The filter gets orientation selectivity by calculating the output of a pool of Difference-of-Gaussians filters, and achieves retinal segmentation by adding up the responses of the two rotation-invariant B-COSFIRE filters and threshold processing. This method got a good segmentation effect for healthy images, but the false positive rate is too high for pathological images. Mendonca et al. [14] realized automatic blood vessel segmentation by combining the extraction of blood vessel centerline and morphological reconstruction. This method is ineffective in the segmentation of micro-vessels and has the mistaken examination of the optic disc, lesions and background. Fraz et al. [15] used center line detection combined with position plane morphological transformation for vessel segmentation. This method is fast and efficient and can suppress noise better, but it does not consider the significant features such as blood vessel profile, and the selection of structural elements is more stringent [16].

### Supervised method

Supervised methods mainly train classifiers based on extracted features to classify non-vessel and vessel. Ricci et al. [17] used line operation combined with support vector machine (SVM) to learn samples. The feature extraction is simple and the required samples are few. Marin et al. [18] proposed a multilayer feed-forward neural network to detect retinal blood vessel. The neural network can be trained on only one database but get good segmentation results on multiple databases. Wang et al. [19] put forward a hierarchical retinal blood vessel segmentation method. Firstly, they used the histogram equalization and Gauss filtering to enhance the green channel, then adopt a simple linear iterative cluster (SLIC) method to segment the super-pixels. Finally, they applied convolutional neural networks (CNN) to extract hierarchical features and classify them with random forests. This kind of surveillance method is to extract the relevant features after getting the corresponding calibrated vascular segmentation results, and then use the classifier to train. Jiang et al. [20] divided the entire image into multiple image patches and proposed a fully convolutional network with transfer learning to segment the retinal blood vessels. The method requires image preprocessing with contrast enhancement, data augmentation, network training and testing, small slices merging and post-processing with de-noising, where pre-processing and post-processing requires human manipulation, thereby increasing subjective factors and consuming time.

These classification models depend on the quality of manual feature selection and need many pre-segmented retinal vessel images as training samples to ensure the accuracy of the model, which requires high requirements for medical images.

Deep learning algorithm has been popular with academic and industry in recent years. It combines shallow features to form abstract deep features, and then discovers the distributed features of data. Compared with traditional methods, deep learning allows computers to learn from observation data and solve problems on their own according to the learning results. Liskowski et al. [21] extracted image patches from large images for data augmentation and used deep neural network for retinal vascular segmentation. Fu et al. [22] transformed the vascular segmentation to a boundary detection problem. The segmentation probability map was generated by holistically nested edge detection (HED), and then the binary segmentation results were obtained by conditional random field (CRF). Khalaf et al. [23] simplified the structure of CNN to distinguish the big vessels, small vessels and background in fundus images, and adjusted the convolution cores of different sizes. Ngo et al. [24] proposed a max-resizing technology to improve network training, which achieved good segmentation effect in DRIVE dataset [25].

Full convolutional network (FCN) [26], as an important branch of deep learning, is proposed based on image semantic segmentation. Ground truth is used as the supervisory information training network, which makes the network predict at the pixel level, and further extends the classification at the image level to the classification at the pixel level. U-Net [27] model is a semantic segmentation network based on FCN, which is suitable for medical image segmentation. The network adopts the structure of encoder and decoder. The spatial dimension of the pooling layer is gradually reduced by the encoder, and the details and spatial dimension of the image are gradually restored by the decoder. In addition, the skip connection between the encoder and decoder is also used to help the decoder repair the details of the target better. Jin et al. [28] proposed DUNet for retinal vessel segmentation in an end to end manner and experimented on DRIVE [25], STARE [29] and CHASE_DB1 [30] dataset. Laibacher et al. [31] improved the traditional U-Net and proposed M2U-net, added the pre-training component of MobileNetV2 in the encoder part, added the new bottleneck block in the decoder part, and integrate with bilinear sampling, reduced the number of parameters greatly. Inspired by the success of ResNet [32] and R2U-Net [33], Zhuang et al. [34] proposed LadderNet for retinal blood vessel. Unlike U-net, LadderNet has many pairs of encoder-decoder branches and skips connections between each pair of adjacent decoders and decoder branches at each level. In addition, LadderNet uses modified residual blocks, in which two convolution layers share the same weight. Gu et al. [35] proposed CE-Net for medical image segmentation which adopted pre-trained ResNet block in the feature encoder and applied dense atrous convolution block and residual multi-kernel pooling in context extractor. Hu et al. [36] proposed a multiscale CNN architecture with an improved cross-entropy loss function and fully connected conditional random field (CRF) to detect hard examples and more details in fundus images. Mo et al. [37] introduced a multi-level deep supervised network to retinal vessel segmentation. This method does not rely on manual features, which reduces the impact of subjective factors. Chen et al. [38] applied prior knowledge to feature learning of deep neural networks and proposed a labeling-free approach for retinal blood vessel segmentation.

Although the existing deep learning method can learn the vessel features by increasing the depth of the network, it is easy to ignore the elongated blood vessel structure, resulting in inconspicuous segmentation results. In addition, most methods have better segmentation results on healthy fundus images while the segmentation performance of lesion images is not desirable.

We are looking forward to getting a retinal vessel segmentation method that overcomes the shortcomings of traditional unsupervised and supervised methods. Therefore, a bidirectional symmetric cascade network is proposed in this paper to achieve accurate vessel segmentation of fundus image.

## Methods

**Formulation** Let (*X*,*Y*) represent the image pair on the training set *T*, which *X*={*x*_*i*_,*i*=1,…,*m*} represents the input fundus image, *Y*={*y*_*i*_,*i*=1,…,*m*,*y*_*i*_∈(0,1))} representing the ground truth corresponding to the fundus image. Since the diameters of the retinal blood vessels are different, the blood vessel edges are decomposed into many binary contour maps according to the width of the retinal image blood vessels, i.e.,

1 $$\begin{array}{@{}rcl@{}} Y=\sum_{d=1}^{D}Y_{d}, \end{array} $$

where *Y*_*d*_ denotes the vessel contour labeling image with diameter *d*.

The goal of this paper is to learn a vessel contour detector *C*(·) capable of detecting different diameters by training deep neural networks. Specifically, this paper needs to build a deep convolutional neural network with *D* convolutional layer, in which different convolution layers can adaptively learn the scale information from each layer to describe the retinal vessel contours of different diameters.

For a training image *X*, assuming that the feature map output by the *d*−*th* convolutional layer is *M*_*d*_(*X*)∈*R*^*l*×*w*×*h*^, *M*_*d*_(*X*) as an input to build a vessel contour detector *C*(·), the loss function of the layer is expressed as

2 $$\begin{array}{@{}rcl@{}} L_{d}=\sum_{X\in{T}}|P_{d}-Y_{d}|, \end{array} $$

where *P*_*d*_=*C*_*d*_(*M*_*d*_(*X*)) represents the prediction results of blood vessel contour with vessel diameter of *d*. Thus, the final vessel contour detector is formulated as the sum of the contour detectors learned from diameter scale 1 to *D*, then the global loss function is formulated as

3 $$\begin{array}{@{}rcl@{}} L_{d}=\sum_{d=1}^{D}(P_{d}-Y_{d}). \end{array} $$

*Y*_*d*_ should be known in advance in order to calculate the loss function. Obviously, it is unrealistic to artificially decompose the ground truth of the retinal image to different diameter scales, which makes it difficult to obtain a blood vessel contour label with a diameter scale of *d*. We consider that the difference between ground truth and other layer contour prediction results can be used to approximate the vessel label *Y*_*d*_ of the specific diameter scale of the *d*−*th* layer, i.e.,

4 $$\begin{array}{@{}rcl@{}} Y_{d}\sim{Y-\sum_{i\neq{d}}P_{i}} \end{array} $$

However, we found that the blood vessel contour label obtained by Eq. (4) does not adaptively learn the diameter scale information that the convolution layer itself can capture after the following proof. According to Eq. (4), for a training image, the blood vessel contour prediction result of *d*−*th* layer approximates the ground truth of the blood vessel contour at layer *d*, i.e., $Y_{d}\sim {Y-\sum _{i\neq {d}}P_{i}}$. The vascular contour prediction results of the previous convolution layers are transmitted to *d*−*th* for training., and get the equivalent formula,i.e., $Y\sim {\sum _{i=1}^{d}P_{i}}$. Then the loss function of Eq. (3) is converted to *L*=*L*(*Y*_∗_,*Y*), where $Y_{*}=\sum _{i=1}^{d}P_{i}$. According to the chain rule, the gradient of blood vessel contour prediction result *P*_*d*_ is

5 $$\begin{array}{@{}rcl@{}} \frac{\partial(L)}{\partial(P_{d})}=\frac{\partial(L(Y^{*},Y))}{\partial(P_{d})}=\frac{\partial(L(Y^{*},Y))}{\partial(Y^{*})}\bullet{\frac{\partial(Y^{*})}{Y_{d}}} \end{array} $$

From Eq. (5), the gradients of the vascular contour prediction results *P*_*i*_ and *P*_*d*_ generate for any two convolutional layers *i* and *d*(*i*≠*d*) are consistent because $\frac {\partial (y^{*})}{\partial (P_{i})}=\frac {\partial (y^{*})}{\partial (P_{d})}=1.$ That is to say, the training process according to Eq. (4) has been supervising each convolution layer with the same label, and it cannot adaptively learn the blood vessel diameter scale information suitable for each layer.

Aiming at the above problem, we decompose the vessel contour label *Y*_*d*_ into two complementary supervisions, one of which ignores vessels with diameter scales smaller than *d* and the other ignores vessels with diameter scales greater than *d*. These two-supervision train two vessel contour detectors on each diameter scale. In fact, the supervision refers to the prediction of the blood vessel contour of each intermediate layer. We define two complementary supervises of the vessel contour label *Y*_*d*_ with diameter scale *d* as

6 $$\begin{array}{@{}rcl@{}} \begin{aligned} Y_{d}^{l2h}=Y-\sum_{i< d}P_{i}^{l2h}\\ Y_{d}^{h2l}=Y-\sum_{i>d}P_{i}^{h2l} \end{aligned} \end{array} $$

The superscript *l*2*h* indicates the information dissemination from low layers to high layers of the network, *h*2*l* indicating the information dissemination from high layers to low layers of the network. For the blood vessel contour prediction results with diameter scale *d*, $P_{d}^{l2h}$ and $P_{d}^{h2l}$ are approximately equal to $Y_{d}^{l2h}$ and $Y_{d}^{h2l}$ respectively, so the sum of the two is similar to *Y*_*d*_, i.e.,

7 $$\begin{array}{@{}rcl@{}} Y_{d}^{l2h}+Y_{d}^{h2l}\sim{2Y-\sum_{i< d}P_{i}^{l2h}-\sum_{i>d}P_{i}^{h2l}} \end{array} $$

Therefore, we use $Y_{d}^{l2h}+Y_{d}^{h2l}$ to represent the blood vessel contour prediction results with diameter scale of *d*.

**Fig. 2:**
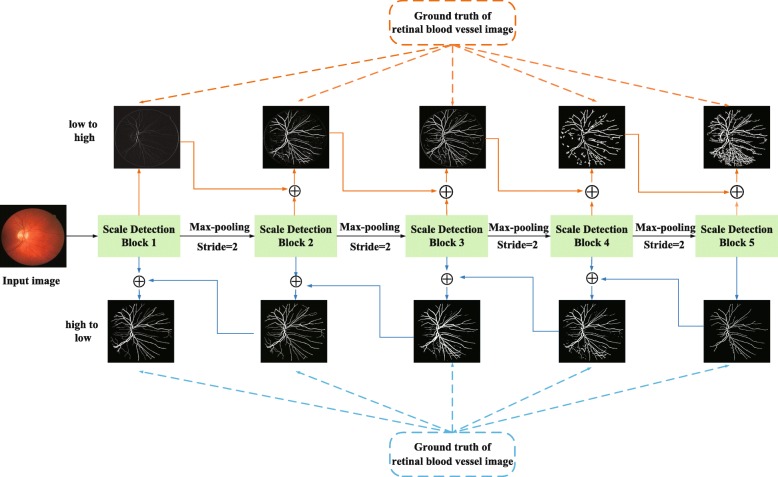
The overall architecture of the bidirectional symmetric cascade network

**Fig. 3:**
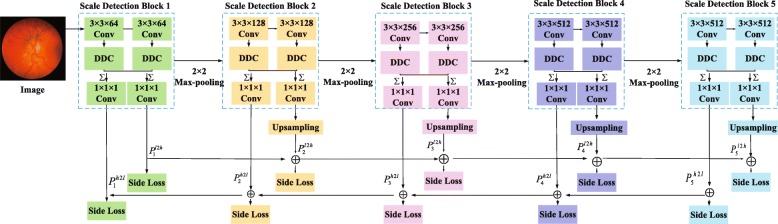
The detailed architecture of the bidirectional symmetric cascade network

**Fig. 4:**
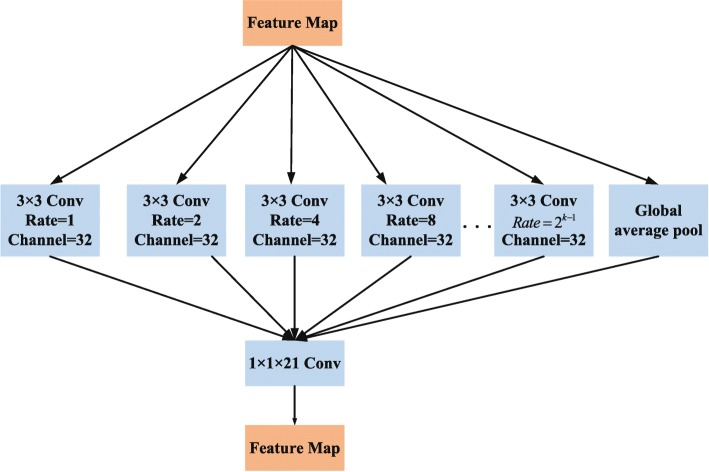
The detailed architecture of the dense dilation convolution module

**Network architecture** According to Eq. (7), we propose a bidirectional symmetric cascade network architecture for retinal blood vessel segmentation. The overall architecture of bidirectional symmetric cascade network is shown in Fig. 2. As shown in Fig. 2, the network framework is composed of five scale detection blocks, and each scale detection blocks generates two different blood vessel contour prediction maps through two paths from lower layers to higher layers and from high layers to low layers. Specifically, the network improves the original VGG 16 [39] by removing the three fully connected layers and the last pooling layer, and then divides the remaining 13 convolutional layers into five VGG blocks, each block followed by a max-pooling layer to increase the receptive field of the next block. Inserting a dense dilation convolutional module after the VGG block to make it a scale detection module. The details of the bidirectional symmetric cascade network and the dense dilated convolution module are shown in Figs. 3 and 4.

**Scale Detection Block** The scale detection block is the basic constituent unit of this network. Each scale detection module ultimately generates two vessel contour prediction maps. As shown in Fig. 3, each scale detection block consists of several convolutional layers, each followed by a dense dilation convolution module. The outputs of the multiple dense dilation convolution modules are fused into two convolutional layers to produce two 1×1 vessel contour prediction results $P_{d}^{l2h}$ and $P_{d}^{h2l}$ respectively. For the *d*−*th* scale detection block, the supervision $Y_{d}^{l2h}$ and $Y_{d}^{h2l}$ calculated by Eq. (6) is used for training $P_{d}^{l2h}$ and $P_{d}^{h2l}$. $P_{1}^{l2h}$ is one of the outputs of the first stage, and resolution of the blood vessel contour prediction map is consistent with the original image, so no upsampling is required. $P_{1}^{l2h}$ is sent to all later stages and is added to the upsampled output of other scale detection modules to compute loss function from low layers to high layers at the current diameter scale. The final blood vessel contour prediction result is calculated by 1×1 convolution fusion of the blood vessel contour prediction map generated by all the intermediate layers.

**Dense Dilated Convolution Module** Inspired by Inception-Resnet [40] and dilated convolution, we propose a dense dilated convolution module. This module after the VGG block to enhance the multi-scale representation of the retinal blood vessel image. A two-dimensional feature map *x*∈*R*^*L*×*H*^ as an input for the convolution filter *w*∈*R*^*l*×*h*^, the dilated convolution output $y\in {R^{L^{'}\times {H^{'}}}}$ at location (*i*,*j*) is

8 $$\begin{array}{@{}rcl@{}} Y_{i,j}=\sum_{p,q}^{l,h}x_{[i+rq,j+rq]}\bullet{w_{p,q}} \end{array} $$

where *r* is the dilation rate, representing the stride of sampling input feature map. Equation (8) can be converted to a standard convolution when *r*=1. Equation (8) shows that the dilation convolution effectively increases the receptive field of neurons without significantly reducing the retinal feature map resolution and increasing the convolutional neural network parameters.

As shown in Fig. 4, each of the dilated convolutions in the dense dilated convolution module is stacked in cascade. This module contains convolution branches with different dilation rates, and the rates are increased from 1, 2, 4 to 2^*k*−1^ respectively. For the *k*−*th* convolution, we set the dilation rate as *r*_*k*_=2^*k*−1^, which *k* represents the number of convolution layers. 1×1 convolution is used for linear activation in each dilated convolution branch. Generally, large receptive fields extract abstract features at the object level, and small receptive fields pay more attention to the details of images. The dense dilated convolution module formed by combining dilated convolution branches of different dilation rates can extract retinal vessel contour features of different diameters for more fine segmentation.

**Loss Function** Since there are two paths from low to high and high to low in the network, each scale detection block has two side loss. In addition, the ten retinal vessel contour prediction maps generated by the five scale detection blocks are fused to get the fusion layer loss. Therefore, the overall loss of the network consists of side loss and fusion layer loss, it can be formulated as

9 $$\begin{array}{@{}rcl@{}} L=w_{side}\bullet{L_{side}}+w_{fuse}\bullet{L_{fuse}(P,Y)} \end{array} $$

10 $$\begin{array}{@{}rcl@{}} L_{side}=\sum_{d=1}^{D}L(P_{d}^{l2h},Y_{d}^{l2h})+L(P_{d}^{h2l},Y_{d}^{h2l}) \end{array} $$

11 $$\begin{array}{@{}rcl@{}} L_{fuse}=|P-Y| \end{array} $$

where *L*_*side*_ and *L*_*fuse*_ represent the weight of the side loss and the fusion layer loss respectively. *P* indicates the final retinal vessel contour prediction map. The loss function *L*(·) is calculated by the difference between the predicted value and label of each pixel in fundus images. There is a great imbalance between vessels and non-vessels, the training result tends to be more prone to non-vessel if not consider the sample balance issue. To solve the potential over-fitting problem, we use a class-balanced cross-entropy function as *L*(·). In addition, since the diameter of the retinal blood vessels is different in width, a threshold *η* is introduced to calculate loss function to divide the positive and negative class. Let a ground truth *Y*={*y*_*i*_,*i*=1,…,*m*,*y*_*i*_∈(0,1))}, *Y*^+^ and *Y*^−^ denotes vessel and non-vessel pixel, we define *Y*^+^={*y*_*i*_|*y*_*i*_>*η*} and *Y*^−^={*y*_*i*_|*y*_*i*_=0},the loss function is defined as

12 $$\begin{array}{@{}rcl@{}} L(Y^{*},Y)=-\beta\sum_{i\in{Y^{+}}}log{y_{i}}-(1-\beta)\sum_{i\in{Y^{-}}}log{(1-y_{i}^{*})} \end{array} $$

where $Y^{*}=\{Y_{i}^{*}|i=1,\dots,m,y_{i}^{*}\in {(0,1)}\}$ denotes retinal blood vessel prediction maps, 1−*β*=*λ*∙|*Y*^+^|/*Y* and *β*=|*Y*^−^|/*Y* are used to balance blood vessel and background class in retinal blood vessel images.

**Fig. 5:**
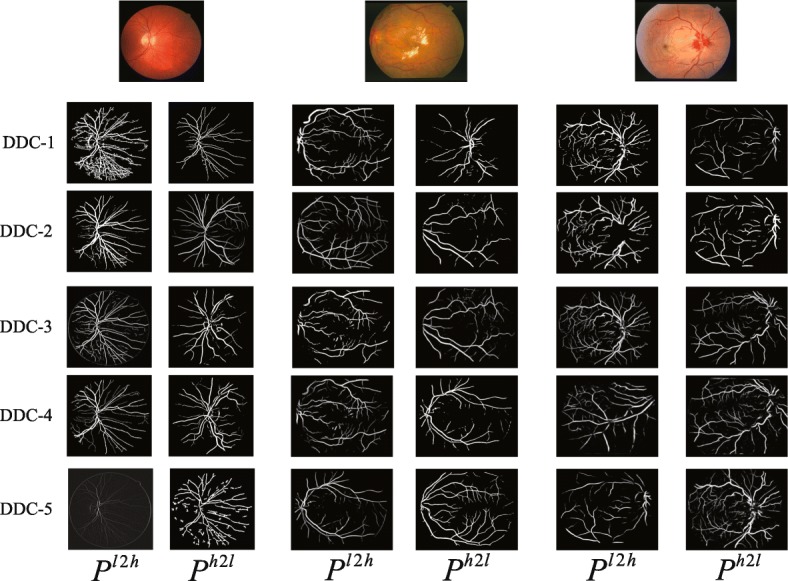
Examples of blood vessel contour detected by different dense dilated convolution module (DDCM). Each DDCM generates two blood vessel contour predictions, *P*^*l*2*h*^ and *P*^*h*2*l*^, respectively

Figure 5 shows the retinal vessel contours detected by different dense scale detection modules. As can be seen from Fig. 5, different dense-scale detection modules are capable of extracting blood vessel information of different diameter scales. From the top to the bottom, the low-level scale detection module pays more attention to the rich local details such as tiny blood vessels, while the high-level scale detection module can extract the general structure of the retinal vessels, and the contours of the vessels with larger diameter scales are more sensitive. Taking the middle image of Fig. 5 as an example, the high-level detection module ignores the fine capillaries around the bright spots, and the low-level detection module can accurately extract them.

## Results

**Datasets** We experiment with our method on the three public datasets: DRIVE, STARE and HRF. The DRIVE dataset [25] is a color fundus library established by the Niemeijer team in 2004 based on the screening of diabetic retinopathy in the Netherlands. There are 7 fundus images of early diabetic retinopathy, and 33 fundus images without diabetic retinopathy. It is divided into training set and test set, each containing 20 images, and the resolution of images is 565×584.

The STARE dataset [29] consists of 20 fundus images of which 10 images have lesions and 10 images without lesions and the image resolution is 605×700. Each image corresponds to two expert manual segmentation results.

The HRF dataset [41] consists of 15 healthy fundus images, 15 diabetic retinopathy fundus images and 15 glaucoma fundus images with a resolution of 3504×2336. Each image corresponds to an expert manual segmentation result. It is the fundus images dataset of highest resolution at present.

The CHASE_DB1 dataset [30] includes 28 retinal images taken from the eyes of 14 schoolchildren, which the first 20 images are used for training, while the remaining 8 images are used for testing. The resolution of each image is 999×960.

**Experiment settings** We experimented with the proposed method on the Ubuntu 18.04 system with NVidia GeForce Titan graphics cards with 16G RAM. This network uses VGG [39] pre-trained on ImageNet as the backbone. The implementation of this network is based on Pytorch platform.

We set the threshold *η* used to calculate the loss function as 0.4 and the parameter *λ* as 1.1. The weights *w*_*side*_ and *w*_*fuse*_ in the loss function are set as 0.5 and 1.2 respectively. We adopt a small batch random gradient descent method to achieve fast convergence of the network in the training stage. The batch size is set to 8, and the initial learning rate, momentum, and weight decay are set as 2*e*^−3^, 0.9 and 10^−4^. In addition, we use the multi-learning strategy to update the learning rate. The learning rate is the initial learning rate multiplied by $\left (1-\frac {iter}{max\_iter}\right)^{power}$, where power is 0.9, the initial learning rate is 2*e*^−3^, and the maximum number of iterations is 150.

**Fig. 6:**
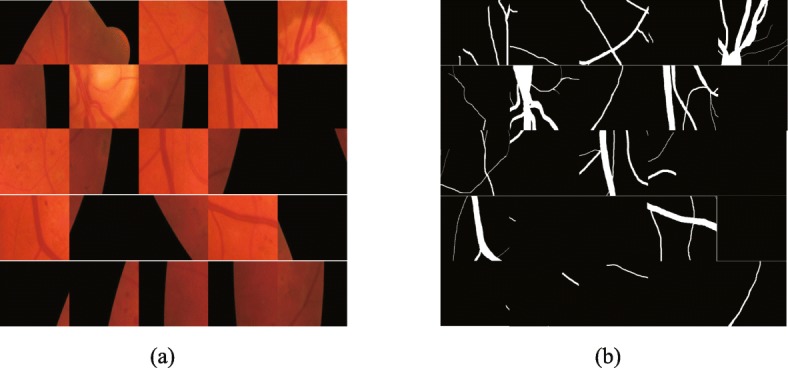
Image patches on DRIVE. **a** patches of the original image **b** ground truth patches corresponding to the original image

Due to the limited image of the DRIVE, STARE and HRF datasets, it is necessary to enhance the original retinal image. Firstly, we crop the retinal image and its corresponding ground truth into 50×50 patches. Since there are 20 images in DRIVE for training and validation, and the remaining 20 images for testing, we randomly select 20,000 patches generated by 20 training/validation set for experiments, of which 180,00 are used for training and 2,000 are used for validation. Some image patches of DRIVE and their corresponding ground truth are shown in Fig. 6.

Secondly, the training set and test set are not explicitly divided for STARE. There are two main strategies for dividing the training set and test set at present. One is to randomly select the image patches of the STARE for training, but it will result in some overlap between the training and test set. Another solution is to leave a validation method that only one image is tested, and the other 19 images for training. There is no sample overlap between the training set and test set. Therefore, we choose the second strategy to STARE. In addition, we crop the retinal image and its corresponding ground truth into 50×50 patches.

Thirdly, we manually divided HRF into 38 training/validation sets and 9 test sets. Besides, we crop the retinal image and its corresponding ground truth into 50×50 patches. During the experiment, we randomly selected 40,000 patches in the training/validation set, of which 36,000 patches for training and 4000 patches for validation.

In addition, we use the following methods to achieve data augmentation. Firstly, changing the contrast of the original image. The saturation component and the luminance component in the HSV color space are changed by exponential transformation, and the exponential coefficient is from -0.6 to 0.8 in steps of 0.2. Secondly, the original image is scaled from 0.75 to 1.05 in steps of 0.05. Thirdly, rotating the original image from to in steps of. Finally, moving the original image. The original retinal image is translated from -80 pixels to 100 pixels in the horizontal and vertical directions, in steps of 20 pixels.

**Evaluation metrics** The goal of retinal vessel segmentation is to get the segmentation result of each pixel and determine whether the pixel is blood vessel or background. By comparing the ground truth (GT) with the segmentation results (SR), there are four cases: True positive (TP), which indicates the number of pixels that correctly divide the blood vessel into positive categories: False Positive (FP), which indicates that the background is misclassified into positive pixels. True Negative (TN), which indicates the number of pixels that divide the blood vessel into negative categories; False Negative (FN), which indicates the number of pixels that correctly segment the background into negative categories.

According to the above four quantitative indicators, there are six evaluation indicators to evaluate the experimental performance: Sensitivity (Se), Specificity (Sp), Accuracy (Acc), Precision (Pr), F-Measure (*F*_1_) and Dice coefficient. Their definitions are as follows:

13 $$\begin{array}{@{}rcl@{}} Se=\frac{TP}{TP+FN} \end{array} $$

14 $$\begin{array}{@{}rcl@{}} Sp=\frac{TN}{TN+FP} \end{array} $$

15 $$\begin{array}{@{}rcl@{}} Acc=\frac{TP+TN}{TN+TP+FN+FP} \end{array} $$

16 $$\begin{array}{@{}rcl@{}} Pr=\frac{TP}{TP+FP} \end{array} $$

17 $$\begin{array}{@{}rcl@{}} F_{1}=\frac{2\times{Pr}\times{Se}}{Pr+Se} \end{array} $$

18 $$\begin{array}{@{}rcl@{}} Dice=\frac{2|GT\bigcap{SR}|}{|GT|+|SR|} \end{array} $$

Sensitivity (Se) indicates the proportion of correctly segmented blood vessel pixels to real blood vessel pixels, and the Specificity (Sp) indicates the proportion of correctly segmented background pixels to the real background pixels. The Accuracy (Acc) indicates the proportion of correctly segmented pixels to the total pixels of the image. Precision indicates the proportion of correctly segmented blood vessel pixels that are predicted to be blood vessel pixels. *F*_1_ is the weighted harmonic mean of Precision and Recall, and the Dice coefficient represents the ratio of the ground truth to the intersection and the union of the predicted segmentation results.

A receiver operating characteristic (ROC) curve is a curve with true positive rate as the ordinate and false positive rate as the abscissa, which can visually indicate the quality of the classifier. The value of AUC is the area under the ROC curve. The value is between 0.5 and 1. The larger the value of AUC, the better the algorithm works.

**Fig. 7:**
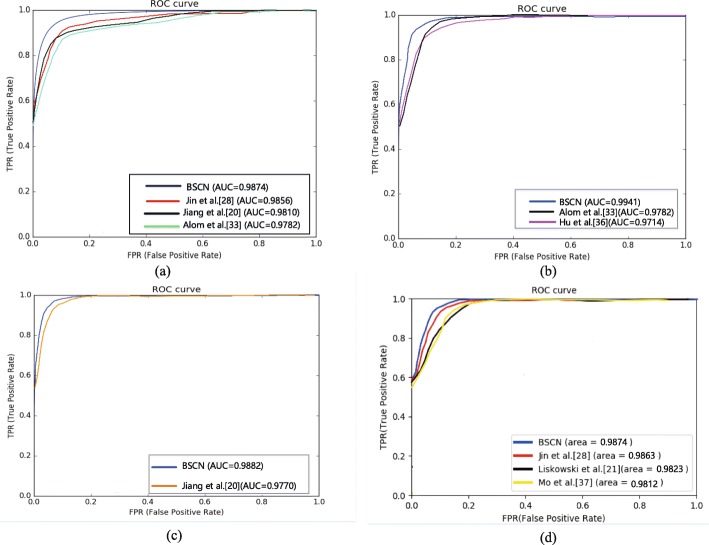
ROC curve of different methods. **a** ROC curve on DRIVE **b** ROC curve on STARE **c** ROC curve on CHASE_DB1 **d** ROC curve on HRF

**Performance of the proposed method** In order to evaluate the effectiveness of the proposed method, Table 1 shows the performance comparison results of the proposed method and the second expert manual segmentation on the DRIVE, STARE, HRF and CHASE_DB1 datasets in evaluation metrics of Se, Sp, Acc and AUC. Table 1 shows that the sensitivity, specificity and accuracy of the proposed method on all four datasets are higher than those of the second expert, indicating that our method has better ability of accurately classify the blood vessel and the background, and has a lower false positive rate than the second expert manual segmentation result. As shown in Fig. 7, the AUC values of our method are more than 0.98 on the three datasets, indicating that the bidirectional symmetric cascade network has better generalization ability.

**Table 1:** Comparison of results between manual segmentation and the proposed method on the DRIVE, STARE, HRF and CHASE_DB1 datasets

Dataset	Method	Se	Sp	Acc	AUC
DRIVE	2nd expert	0.7760	0.9725	0.9473	-
	Proposed Method	0.8179	0.9879	0.9846	0.9874
STARE	2nd expert	0.8719	0.9388	0.9353	-
	Proposed Method	0.8751	0.9894	0.9872	30.9941
HRF	2nd expert	0.8010	0.8011	0.9650	-
	Proposed Method	0.8025	0.9854	0.9856	0.9882
CHASE_DB1	2nd expert	0.7686	0.9779	0.9560	-
	Proposed Method	0.7972	0.7972	0.9889	0.9874

**Comparison with the state-Of-The-Art-Methods**

**Performance On DRIVE**

**Fig. 8:**
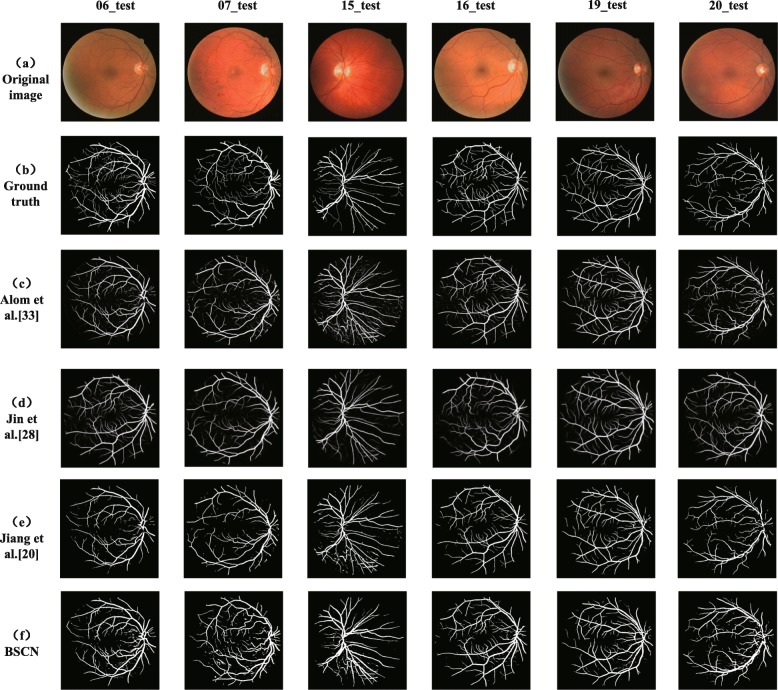
Qualitative results comparison of different methods on DRIVE dataset

We compared the experimental results of the proposed method with existing methods on the DRIVE dataset, and the segmentation results are shown in Fig. 8. It can be seen from Fig. 8 that compared with the other three models, the proposed method is better for segmentation of tiny blood vessels which are not easy to identify. R2U-Net proposed by Alom et al. [33] is an end-to-end network architecture that includes an encoder and a decoder. However, some blood vessel detail information lost due to downsampling in the encoding process, so that the small blood vessels in the retinal image are not segmented. Jiang et al. [20] transformed the traditional whole image segmentation problem into regional semantic element segmentation task, and proposed a full convolutional neural network with transfer learning method to achieve blood vessel segmentation. However, the pre-training semantic segmentation model of AlexNet is not sensitive to small objects, while the manually labeled ground truth images in the DRIVE dataset contains abundant capillaries, which results in the sensitivity of the method is not high enough to successfully segment the tiny blood vessels in the funds image. Taking the 06_test image for example, due to the low contrast between the blood vessels and the background, and the rich capillaries in this image, it is difficult to completely segment some fine blood vessel distal ends. Although DUNet proposed by Jin et al. [28] can detect thick blood vessel contours, it cannot accurately segment the branches of blood vessels in the intricate intersection of blood vessels. However, the BSCN proposed in this paper has multiple dense dilated convolution modules, which can adaptively extract retinal vessel features of different diameters by dilated convolution with different dilation rates. Therefore, the segmentation result is more accurate. As can be seen from the sixth line of Fig. 8, our method can not only segment the thick blood vessels but also recognize the tiny blood vessels even difficult to distinguish with eyes. This is not possible with the above three models.

**Fig. 9:**
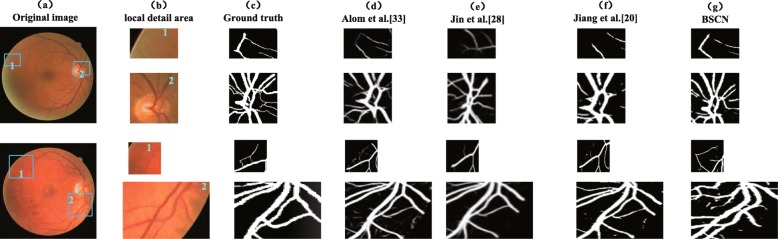
Local detail results comparison of different methods on DRIVE dataset

In addition, Fig. 9 shows segmentation results of DRIVE in local detail areas. The first row shows the low-contrast fine blood vessels, and the second row shows the blood vessels at the intersection. As shown in the first row of Fig.‘9, the method proposed by Jiang et al. [20], Jin et al. [28] and Alom et al. [33] fail to capture the contour information of the tiny capillaries around the optic disc, but our method adaptively captures the retinal blood vessel contours of different diameters by using multiple scale detection blocks to segment the low-contrast tiny blood vessels. As we can see from the second row of Fig. 9, the model proposed by Jiang et al. [20], Jin et al. [28] and Alom et al. [33] can only extract rough blood vessel contour information at the intersection of multiple blood vessels that are close to each other, and our method can capture various diameter of retinal blood vessels and successfully segment the blood vessels that seem to be entangled but actually separated with the help of the dense dilation convolution module. The experimental results in Fig. 9 show that our method is better than the other three methods in the case of low-contrast fine blood vessels and complex interlaced vascular trees, and can achieve better results.

Table 2 compares the quantitative result of our method against the state-of-art methods in evaluation metrics. As shown in Table 2, the proposed method reaches 0.8179, 0.9879, 0.9846, 0.8667 and 0.9874 on Se, Sp, ACC, Pr and AUC, respectively, which are 0.0285, 0.0009, 0.0149, 0.0130, 0.0018 higher than those proposed by Jin et al. [28].

**Table 2:** Quantitative result comparison of different models on the DRIVE

Method	Se	Sp	Acc	Pr	*F*_1_	Dice	AUC
Zhao et al. [11]	0.7420	0.9820	0.9540	-	-	-	0.8620
Azzopardi et al. [13]	0.7716	0.9710	0.9497	-	-	-	0.9563
Fraz et al. [15]	0.7152	0.9768	0.9430	-	-	-	-
Jiang et al. [20]	0.7540	0.9825	0.9624	-	-	-	0.9810
Liskowski et al. [21]	0.7569	0.9816	0.9533	-	-	-	0.9744
Fu et al. [22]	0.7294	-	0.9470	-	-	-	
Jin et al. [28]	0.7894	0.9870	0.9697	0.8537	-	-	0.9856
Laibacher et al. [31]	-	-	0.9630	-	-	0.8006	0.9714
Alom et al. [33]	0.8108	0.9871	0.9706	-	0.8155	-	0.9782
Zhuang[34]	0.7856	0.9810	0.9561	-	0.8202	-	0.9793
Hu et al. [36]	0.7772	0.9793	0.9533	-	-	-	0.9759
Mo et al. [37]	0.7779	0.9780	0.9521	-	-	-	0.9782
Chen et al. [38]	0.7295	0.9696	0.9449	-	-	-	0.9557
Proposed method	0.8179	0.9879	0.9846	0.8667	0.8236	0.8105	0.9874

**Fig. 10:**
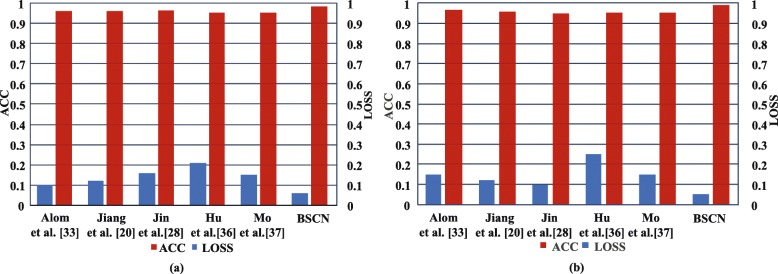
Accuracy and loss results comparison of different method on DRIVE dataset. **a** training set results on DRIVE **b** validation set results on DRIVE

Figure 10 shows the accuracy and loss comparison results for the training and validation sets of the different models on the DRIVE dataset. As shown in Fig. 10, compared with other methods, the BSCN has higher accuracy and lower loss in training and validation phase compared with other methods. In the training phase, the accuracy rate increased by 1.49%, while the loss was reduced by 12% compared to Jin et al. [28]. In the validation phase, the accuracy rate increased by 1.87%, and the loss was reduced by 11% compared to Jin et al. [28].

In addition, we use the ROC curve to evaluate the different methods. The ROC curve on DRIVE is shown in Fig. 7a. The closer the ROC curve is to the upper left boundary, the more accurate the model is trained. It can be seen from Fig. 7a that the ROC curve of the BSCN is the curve of the top left corner of the four models, and the curve of Alom et al. [33] is the lowest of the four curves. The data in the lower right corner of Fig. 7a shows that the area under the ROC curve of the BSCN is the largest, followed by Jin et al. [28], and Alom et al. [33] is the smallest.

**Fig. 11:**
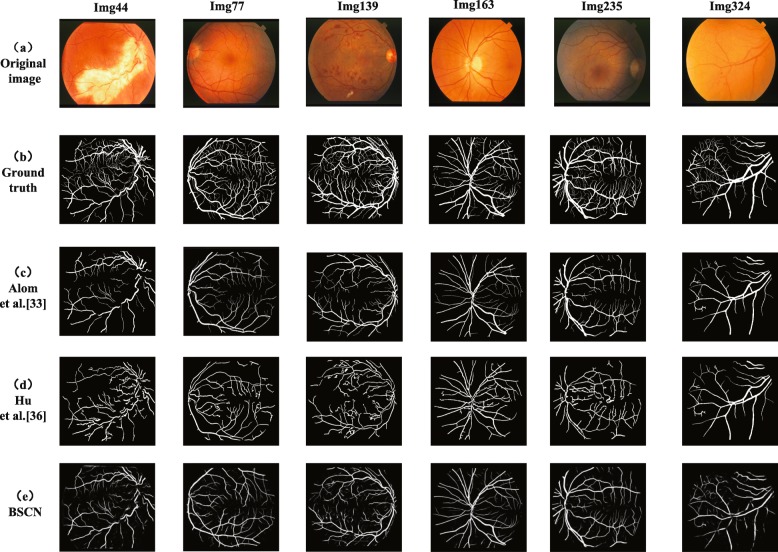
Qualitative results comparison of different methods on STARE dataset

**Performance On STARE** The STARE dataset contains 10 fundus images with different lesions and 10 healthy fundus images. To demonstrate the validity of the proposed method, Fig. 11 displays the comparison of the experimental results of different models on 4 lesion images and 2 healthy images. Image 44 is a fundus image with retinitis, and the bright spots produce significant background differences that result in many blood vessels being discontinuous. The R2U-Net method proposed by Alom et al. [33] lost some vascular context features in the coding process, and thus failed to segment the discontinuous blood vessels. The convolutional neural network and the fully connected condition method proposed by Hu et al. [36] has a poor smoothing ability on the bright spot, which makes the segmentation results more disturbed by noise and fails to segment the blood vessel accurately. The scale detection module in our method has two feature propagation paths from the low layer to the high layer and from the high layer to the low layer, which can generate blood vessel contour prediction maps of different scales, effectively eliminating the influence of the bright spot background and segment discontinuous blood effectively.

Image 77 is a fundus image with hypertensive Retinopathy. Image 139 is a fundus image of the diabetic retinopathy background, and the bright and dark spots in the image greatly interfere with the process of vessel segmentation. Image 324 is a fundus image of hollenhorst plaque, which is difficult to identify the very tiny blood vessel. The method proposed by Alom et al. [33] can only detect the general contour of a blood vessel, but does not recognize small blood vessels. Although Hu et al. [36] can segment the fine blood vessels, it cannot avoid the influence of noise such as vascular discontinuity due to background differences. Moreover, this method is so sensitive to vessel-like lesions and vessels that easy to be disturbed by the lesion and mistakenly recognize the lesions as blood vessels, resulting in inaccurate segmentation results. The proposed method can not only segment small blood vessels but also reduce the false positive rate. Images 163 and 235 are normal fundus images. Alom et al. [33], Hu et al. [36] and our method are capable of segmenting thick and slender blood vessels of healthy images.

Therefore, Fig. 11 shows that for the normal fundus image, the segmentation performance of our method and the other two methods are not much different. For the lesion images, the methods proposed by Alom et al. [33] and Hu et al. [36] are susceptible to interference from the lesion background. However, the robustness of the proposed method is strong, and the blood vessel can be accurately segmented under the condition of different disease background interference.

**Fig. 12:**
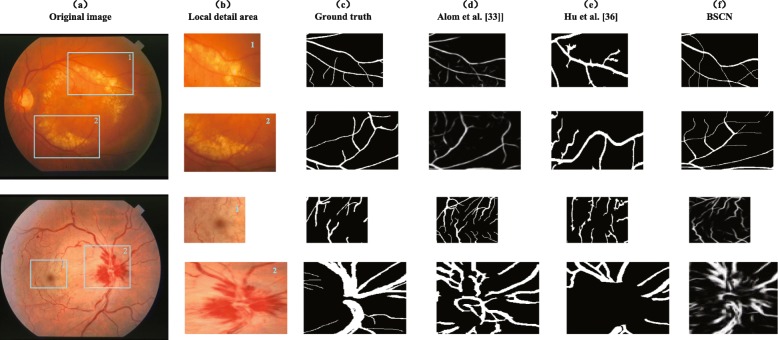
Local detail results comparison of different methods on STARE dataset

Figure 12 shows the comparison of the experimental results of the three methods in local detail areas. Sample 1 is a fundus image with arteriosclerotic retinopathy, and a few bright spots easily interfere with the blood vessel segmentation process. We select the local area around the bright spot to analysis the segmentation effects. As shown in Fig. 12, the R2U-Net method proposed by Alom et al. [33] cannot smooth out the effect of highlight areas on vessel segmentation, only the blurred contour of thick blood vessels around the bright spot can be detected, ignoring the existence of tiny blood vessels. The method proposed by Hu et al. [36] is less robust, and it is easy to misjudge the noise of bright spots as blood vessels, resulting in inaccurate blood vessel segmentation. Sample 2 is a fundus image with central retinal artery and vein occlusion with congestion around the optic disc. We selected dark spots and areas around congestion for comparison. Dark spots and congestion as the background of the lesions caused the blood vessels to discontinue, increasing the difficulty of segmentation of the blood vessels. The method proposed by Alom et al. [33] has the under-segmentation problem, and it cannot identify discontinuous blood vessels that are interrupted by the lesion background so that the sensitivity is reduced. The method proposed by Hu et al. [36] is over-segment to the blood vessel, and the background of lesions such as congestion is mistakenly identified as blood vessels, which increases false positives. However, our method can filter out the influence of background noise on the blood vessels around the optic disc, thus segmenting the discontinuous blood vessels accurately.

Table 3 compares qualitative results of our model against some existing methods in evaluation indicators. As shown in Table 3, our method reaches 0.8751, 0.9894, 0.9872, 0.8547, and 0.9941 on Se, Sp, ACC, F1, and AUC, respectively. Compared with the method proposed by Alom et al. [33], the evaluation results are improved by 0.0643, 0.0023, 0.0166, 0.0151 and 0.0032, respectively.

**Table 3:** Quantitative result comparison of different models on the STARE

Method	Se	Sp	Acc	Pr	*F*_1_	AUC
Zhao et al. [11]	0.7800	0.9780	0.9560	-	-	0.8700
Azzopardi et al. [13]	0.7716	0.9701	0.9497	-	-	0.9563
Fraz et al. [15]	0.7311	0.9680	0.9442	-	-	-
Wang et al. [19]	0.8104	0.9791	0.9621	-	-	0.9751
Jiang et al. [20]	0.8352	0.9846	0.9734	-	-	0.9900
Liskowski et al. [21]	0.8554	0.9862	0.9729	-	-	0.9928
Fu et al. [22]	0.7140	-	0.9536	-	-	-
Jin et al. [28]	0.7428	0.9920	0.9729	0.8856	-	0.9868
Alom et al. [33]	0.8108	0.9871	0.9706	-	0.8396	0.9909
Hu et al. [36]	0.7543	0.9814	0.9632	-	-	0.9751
Mo et al. [37]	0.8147	0.9844	0.9674	-	-	0.9885
Proposed method	0.8751	0.9894	0.9872	0.9856	0.8547	0.9941

**Fig. 13:**
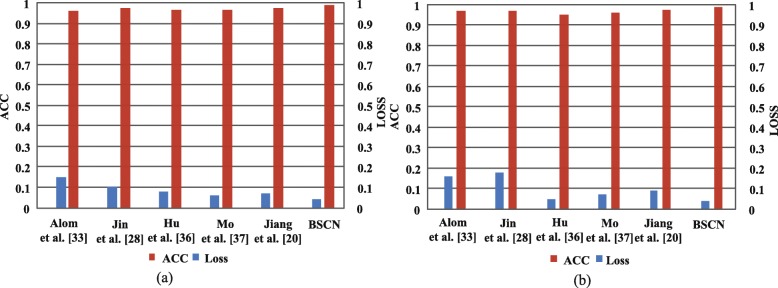
ACC and LOSS results comparison of different method on STARE dataset. **a** training set results on STARE **b** validation set results on STARE

Figure 13a and b show the accuracy and loss results comparison of our method and other existing methods in the training and validation sets of the STARE dataset, respectively. It can be seen from the histogram that compared with other methods, the bidirectional symmetric cascade network proposed in this paper has higher accuracy and lower loss in the training and the validation stage. The accuracy rate increased by 2.75%, while the loss decreased by 11% compared to R2U-Net [33] in the training stage. The accuracy increased by 2.5% while the loss was reduced by 12% during the validation phase compared to R2U-Net [33].

Furthermore, we use the ROC curve to evaluate the experimental method. The ROC curve on STARE is shown in Fig. 7b. It can be seen from Fig. 7b that the ROC curve of the BSCN is the curve of the top left corner of the three models, and the curve of Hu et al. [36] is the lowest of the three curves. The data in the lower right corner of Fig. 7b shows that the area under the ROC curve of the BSCN is the largest, followed by Alom et al. [33], and Hu et al. [36] is the smallest. The above data indicates that the model trained using the BSCN is more accurate.

**Fig. 14:**
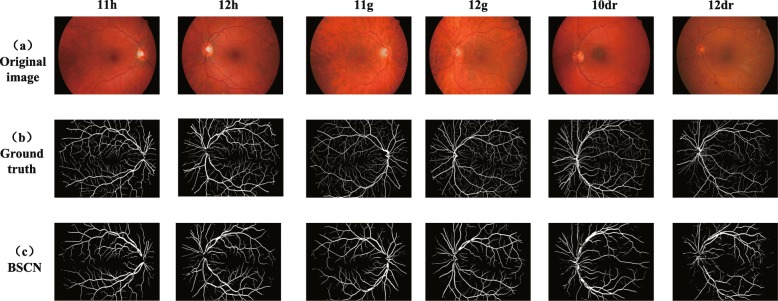
Qualitative results on HRF dataset

**Performance On HRF** Since current researchers rarely conduct the experiment on HRF datasets, we only find Jiang et al. [20] provide experimental quantitative results without providing source code in the paper. So we cannot reproduce the algorithm for experimental verification. Therefore, Fig. 14 only compares the ground truth with the experimental results of our method. The images 11h and 12h are healthy fundus images, and the images 11g and 12g are glaucoma fundus images. The images 10dr and 12dr are fundus images of diabetic retinopathy. It can be seen from the Fig. 14 that whether it is a healthy image or a lesion image interfered by dark spots, our method can eliminate noise interference and accurately segment the fine capillaries, and the experimental effect is good.

Table 4 shows the performance comparison of the method proposed by Jiang et al. [20] and our method. As shown in Table 4, the proposed method reaches 0.8025, 0.9854, 0.9856 and 0.9882 on Se, Sp, Acc and AUC, respectively, which are 3.39%, 0.28%, 1.94% and 1.12% higher than those proposed by Jiang et al. [20].

**Table 4:** Quantitative result comparison of different models on the HRF dataset

Method	Se	Sp	Acc	AUC
Jiang et al. [20]	0.7686	0.9826	0.9662	0.9770
Proposed	0.8025	0.97854	0.9856	0.9882

Figure 7c shows the ROC curve of the different methods on HRF. As shown in Fig. 7c that the ROC curve of the BSCN is the curve of the top left corner, and the curve of Jiang et al. [20] is the lowest. The data in the lower right corner of Fig. 7c shows that the area under the ROC curve of the BSCN is the largest, and Jiang et al. [20] is the smallest.

**Fig. 15:**
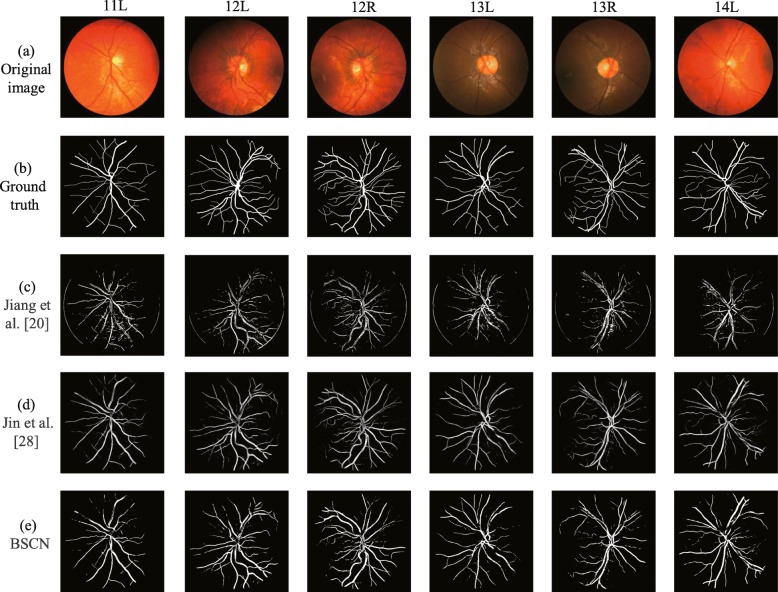
Qualitative results comparion of different methods on CHASE_DB1 dataset

**Performance On CHASE_DB1** Figure 15 presents the experimental results comparison of the proposed method with the state of art methods on the CHASE_DB1 dataset. It can be seen from the original image in the first row of Fig. 15 that the contrast of the blood vessel and the background of the fundus image is low, and there is a problem of significant noise and uneven illumination, which undoubtedly brings difficulties to blood vessel segmentation. As can be seen from the third, fourth and fifth rows of Fig. 15, the proposed method can overcome the above problems and achieves accurate segmentation of tiny blood vessels when compared with the other two methods. Jiang et al. [20] proposed combining the fully convolutional network with transfer learning to achieve vessel segmentation, however the images of different datasets are different, when transfer the model trained on the DRIVE or STARE dataset to CHASE_DB1 cannot overcome the adverse effect of dark spots and resulting in some noise in segment result. Although DUnet proposed by Jin et al. [28] can segment blood vessels of different shapes, it is difficult to avoid the influence of uneven illumination, and the cross vessels at the highlight disc cannot be accurately segmented. However, the bidirectional symmetric cascade network proposed in this paper adaptively extracts retinal vascular features of different diameters by dilated convolution with different dilation rates, and can actually segment the blood vessel of different thicknesses in the case of uneven illumination and low contrast.

Table 5 compares the quantitative result of our method against the state-of-art methods in evaluation metrics. As shown in Table 5, the proposed method reaches 0.7972, 0.9896, 0.9889, 0.8205 and 0.9874 on Se, Sp, ACC, Pr and AUC, respectively, which are 0.0643, 0.0023, 0.0028, 0.1and 0.0032 higher than those proposed by Jin et al. [28].

**Table 5:** Quantitative result comparison of different models on the CHASE_DB1

Method	Se	Sp	Acc	Pr	*F*_1_	Dice	AUC
Azzopardi et al. [13]	0.7585	0.9587	0.9387	-	-	-	0.9487
Fraz et al. [15]	0.7224	0.9711	0.9469	-	-	-	0.9712
Li et al. [9]	0.7507	0.9793	0.9581	-	-	-	0.9716
Jiang et al. [20]	0.8640	0.9745	0.9668	-	-	-	0.9810
Liskowski et al. [21]	0.7816	0.9836	0.9826	-	-	-	0.9823
Jin et al. [28]	0.8229	0.9821	0.9724	0.7510	-	-	0.9863
Alom et al. [33]	0.7459	0.9836	0.9622	-	0.7810	-	0.9803
Laibacher et al. [31]	-	-	0.9703	-	-	0.8006	0.9666
Mo et al. [37]	0.8147	0.9844	0.9674	-	-	-	0.9885
Proposed method	0.7972	0.9896	0.9889	0.8205	0.7560	0.8352	0.9874

**Fig. 16:**
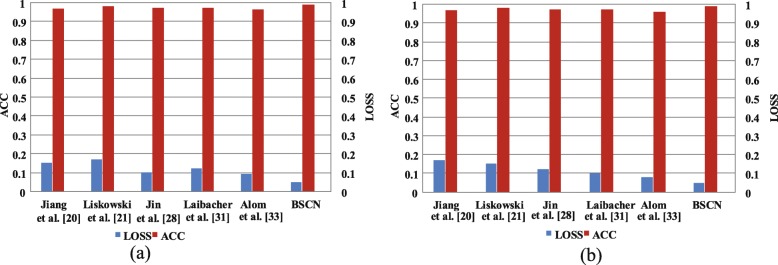
ACC and LOSS results comparison of different method on CHASE_DB1 dataset. **a** training set results on CHASE_DB1 **b** validation set results on CHASE_DB1

Figure 16 shows the accuracy and loss comparison results for the training and validation sets of the different models on the CHASE_DB1 dataset. As shown in Fig. 16, compared with other methods, the BSCN has higher accuracy and lower loss in training and validation phase compared with other methods. In the training phase, the accuracy rate increased by 2.79%, while the loss was reduced by 12% compared to Alom et al. [33]. In the validation phase, the accuracy rate increased by 3.01%, and the loss was reduced by 13% compared to Alom et al. [33].

Furthermore, we use the ROC curve to evaluate the different method. The ROC curve on is shown in Fig. 7d. It can be seen from Fig. 7d that the ROC curve of the BSCN is the curve of the top left corner of the three models, and the curve of Mo et al. [37] is the lowest of the four curves. The data in the lower right corner of Fig. 7d shows that the area under the ROC curve of the BSCN is the largest, followed by Jin et al. [28], and Mo et al. [37] is the smallest. The above data indicates that the model trained using the BSCN is more accurate.

**Ablation study**

In order to verify the contribution of each part in our method, this section performs ablation study. Compared to the other three datasets, the DRIVE dataset has a clear training set and validation set, so the following ablation study is performed on the DRIVE dataset. First, we verify the impact of the layer number *k* of dilated convolutions in the dense dilated convolution module. As shown in Table 6, as the number of dilated convolution layers increases, the number of dilated convolution branches with different dilation rates increases. The dilated convolution becomes a normal convolution, and the input feature map is consistent with the resolution of the output feature map when *k*=1. The performance of the network to detect retinal contours can be significantly improved when *k*>1. However, it cannot be always increased. If is too large, it could not improve the performance of the network, but reduce the segmentation accuracy. Therefore, we set *k*=5 in order to achieve the best experimental results.

**Table 6:** Effect of parameters to vessel segmentation results on the DRIVE

*k*	rate	Acc	AUC	*F*_1_
1	1	0.9817	0.9804	0.8196
2	1,2	0.9825	0.97826	0.8207
3	1,2,4	0.9831	0.9837	0.8211
4	1,2,4,8	0.9846	0.9874	0.8236
5	1,2,4,8,16	0.9846	0.9874	0.8236
6	1,2,4,8,16,32	0.9839	0.9865	0.8229

In addition, we compared different cascaded architectures. Table 7 shows the unidirectional cascade from low layer to high layer (L2H), the unidirectional cascade (H2L) from high layer to low layer, bidirectional cascade (L2H+H2L) and the benchmark, where the benchmark refers to the VGG16 network removing the last three fully connected and pooling layers. It can be seen from Table 7 that the experimental performance using only L2H or H2L is superior to the benchmark. This indicates that the cascade structure is sufficiently effective in the network. The bidirectional cascade structure which combines L2H with H2L structures gets the highest evaluation score, and thus the experimental performance is the best.

**Table 7:** Verify different cascade networks in our method on DRIVE

Architecture	Acc	AUC	*F*_1_
benchmark	0.9807	0.9844	0.8123
L2H	0.9822	0.9853	0.8147
H2L	0.9816	0.9861	0.8125
H2L+L2H(BSCN w/o DDCM)	0.9836	0.9872	0.8243

In Table 8, we further verify the validity of the dense dilated convolution module (DDCM) and the bidirectional cascade structure composed of L2H and H2L. The experimental results show that both the DDCM and bi-directional cascade structure are superior to the benchmark, Acc, AUC and F1 score are increased from 0.9825, 0.9856 and 0.8217 to 0.9836/0.9842, 0.9864/0.9869, 0.8225/0.8239, respectively. The experimental performance of BSCN is the best, and Acc, AUC and F1 are reached 0.9847, 0.9875, and 0.8246, respectively. The above ablation study shows that each component of the proposed method contributes to different degrees of retinal vessel contour detection, and combining bidirectional cascading with dense dilated convolution modules gets the best experimental results.

**Table 8:** Verify DDCM and bidirectional cascade structure in our method on DRIVE

Method	Acc	AUC	*F*_1_
benchmark	0.9825	0.9856	0.8217
DDCM	0.9836	0.9864	0.8225
H2L+L2H(BSCN w/o DDCM)	0.9842	0.9869	0.8239
BSCN	0.9847	0.9875	0.8246

**Computation time**

The proposed method requires 10 h of training on a single Nvidia GeForce Titan GPU. it takes 0.3 s to segment a resolution fundus image. Table 9 shows the average computation time comparison of different models for retinal image vessel segmentation. The DUNet [28], M2U-Net [31] and R2U-Net [33] all use the encoder and the decoder structure to achieve vessel segmentation, while the downsampling and upsampling process produce lots of computational redundancy. Our method produces two blood vessel contour prediction maps from the high-level to the low-level and low-level to high-level paths in each scale detection block. The final blood vessel segmentation results are calculated by convolution fusion of the blood vessel contour prediction maps generated by all the intermediate layers. The computational redundancy of overlapping region features is reduced, thereby reducing computation time. As shown in Table 9, the average computation time of the proposed method is the shortest, which is 21.4 times faster than the calculation time of Alom et al. [33].

**Table 9:** Average computation time comparison for segmenting an image

Type	Method	Computation time
Unsupervised	Azzopardi et al. [13]	10s
	Staal et al. [42]	1.5min
Patch-based supervised	Tan et al. [43]	2.8s
	Liskowski et al. [21]	92s
	Jin et al. [28]	5.8s
	Alom et al. [33]	6.42s
	Ours	0.3s
Image-based supervised	Hu et al. [36]	1.1s
	Fu et al. [44]	1.3s

## Discussion

The previous sections have introduced the network architecture and experimental results of the proposed method, this section will discuss why the BSCD is superior to other networks for retinal vessel segmentation.

In order to obtain more multi-scale features of retinal vessels, some researchers have used very deep networks such as ResNet50 [32] as the backbone framework for vascular segmentation. However, deep networks tend to have more parameters, making the network difficult to train and predicting higher costs. Other researchers have proposed vascular segmentation by constructing image pyramids and incorporating multiple levels of features, which leads to computational redundancy. The previous CNN training strategy for vessel segmentation was to supervise different network layers using a generic ground truth of retinal vessels. However, different network layers can obtain feature information of different scales, so it is not optimal to use the same supervision to train different network layers. In other words, the previous CNN method forced each layer of CNN to predict the vessel contours of all diameter scales, ignoring that a particular intermediate layer only focused on vessel features of certain diameter scales.

In order to avoid the problems of other CNN methods, this paper first improves the original VGG16 by removing the three fully connected layers and the last pooling layer, and then divides the remaining 13 convolutional layers into five VGG blocks, each block followed by a max-pooling layer to increase the receptive field of the next block, which uses a lightweight network structure for retinal vessel contour detection. Secondly, in order to increase the multi-scale feature representation of retinal blood vessels, the Dense Dilated Convolution Module (DDCM) proposed in this paper extracts the retinal vascular features of different diameters by adjusting the dilation rates in the dilated convolution branches, generating two vessel contour prediction results from two directions respectively. The outputs of all the dilated convolution modules are fused to obtain the final blood vessel segmentation results. Thirdly, each layer in the BDCN is supervised by vessel contour label of a specific diameter scale, rather than using a common ground truth to train different layers of the network, optimizing the training process and avoiding computational redundancy.

**Fig. 17:**
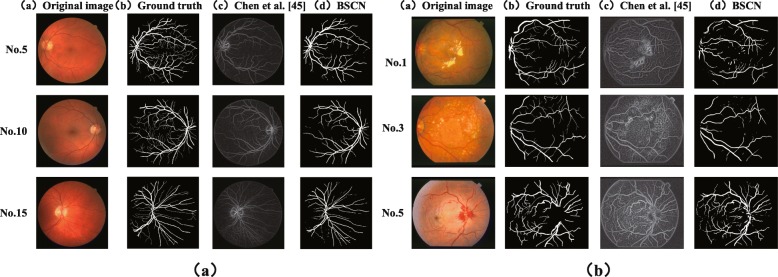
Experimental results with VE-MSC [45] and proposed method on DRIVE and STARE datasets. **a** results on DRIVE **b** results on SATRE

In addition to comparison with convolution neural network methods, it is also compared with traditional methods. Vessel Enhancement via Multi-dictionary and Sparse Coding (VE-MSC) method proposed by Chen et al. [45] obtains a representation dictionary and an enhancement dictionary by extracting patches in the original blood vessel images and label images. The representation dictionary is used to obtain the sparse coefficients, and then the vascular enhancement image is reconstructed by the sparse coefficients and the enhancement dictionary. The theoretical basis of this method is strong, which can effectively improve the image contrast and enhance the detailed information of blood vessels, but the generation of the representation dictionary and enhancement dictionary depends on the selection of image patches. Once the image patch size selection is unreasonable, the corresponding vascular enhancement results may become unsatisfactory. Figure 17 shows the experimental results comparison on the DRIVE and STARE datasets using Chen at al. [45] and our method. As can be seen from the Fig. 17, Chen at al. [45] ignores the diameter scale information of the blood vessels, making the originally fine blood vessels become thicker after being enhanced. In addition, there are lesions and optic disc information showed in experimental results, indicating that the method is susceptible to noise such as the background of the lesion. The dense dilated convolution module proposed in this paper extracts the retinal vessel features of different diameters by adjusting the dilated rate in the dilated convolution branch and generates two blood vessel contour prediction results from two directions respectively. Our method can accurately segment the thick and fine blood vessels and avoid the adverse interference of the lesion background.

Furthermore, the Minimal Path Propagation with Backtracking (MPP-BT) approach proposed by Chen et al. [46] first used the Dijkstra algorithm to start the minimal path propagation from the initial point. The cost value is calculated for each grid point that the propagation front arrives, and then traced from each grid point to the starting point based on the connection information obtained in the previous minimum path propagation. If the starting point is reached before the specified step, the backtracking is stopped. This method can solve the exiting endpoint problem, shortcut problem and accumulation problems, and the information accumulation in the backtracking process can be effectively applied to the breakpoint connection and the construction termination criterion to improve the blood vessel extraction ability. However, this method does not apply to curve structures with significant differences. For example, it will mistake high-contrast edges or artifacts as a curve structure. Early stop propagation may be happening when blood vessels contains a highly complex topology. In addition, this method requires manual selection of the initial point, which undoubtedly increases the initiative of the algorithm. However, each layer in the symmetric bidirectional cascade network proposed in this paper is supervised by the vessel contour labels of specific diameter scale, without determining the initial seed point, which improves the autonomy of the algorithm.

## Conclusions

Aiming at the problem that the previous CNN-based vessel segmentation method is difficult to accurately segment the tiny blood vessels and is susceptible to lesion interference, this paper proposes a bidirectional symmetric cascade network to achieve accurate blood vessels segmentation in retinal images. The bidirectional symmetric cascade network is composed of five scale detection blocks, and each of the two scale detection blocks is connected by a max-pooling layer. In order to fully learn the multi-scale features of retinal vessels to segment retinal vessels of different widths, this paper proposes the dense dilated convolution module. The module extracts retinal vessel features of different diameters by changing the dilation rate, and generates two blood vessel contour prediction results from the low layer to the high layer and the high layer to the low layer of the network respectively. In addition, the proposed method overcomes the problem that the segmentation result is not ideal by using only one common ground truth to train different network layers, and the specific layer supervision is used to train each network layer, allow each layer to focus more on the specific scale of vascular features that it extracts. We performed experiments on the DRIVE, STARE, HRF and CHASE_DB1 datasets. The experimental results show that compared with other methods, our method can not only exclude the lesion interference, but also accurately segment the fine blood vessels in the retina, and the computation time is shorter.

In order to more effectively balance the vascular and non-vascular class differences, we are working on a more efficient loss function to achieve more accurate blood vessel segmentation in the future. In addition, we plan to extend the BSCN framework to the 3D domain to achieve accurate segmentation of 3D medical images.

## Data Availability

Data related to the current study are available from the corresponding author on reasonable request.

## Abbreviations

Acc: Accuracy
AUC: Area under curve
BSCN: Bidirectional symmetric cascade network
CNN: Convolutional neural networks
CRF: Conditional random field
DDCM: Dense dilated convolution module
DRIVE: Digital retinal images for vessel extraction
*F*_1_: F-measure
FCN: Full convolutional network
FN: False negative
GT: Ground truth
H2L: High to low
HED: Holistically nested edge detection
HRF: High-resolution fundus image database
HSV: Hue, saturation, value
L2H: Low to Ligh
MPP-BT: Minimal path propagation with backtracking
Pr: Precision
ROC: Receiver operating characteristic
SDB: Scale detection block
Se: Sensitivity
SLIC: Simple linear iterative cluster
Sp: Specificity
SR: Segmentation results
STARE: STructured analysis of the retina
SVM: Support vector machine
TN: True negative
TP: True positive
VE-MSC: Multi-dictionary and sparse coding
VGG: Visual geometry group
